# Comorbid SUNCT Syndrome and Opalski Syndrome Caused by Dorsolateral Medullary Infarction

**DOI:** 10.3389/fneur.2020.00052

**Published:** 2020-02-11

**Authors:** Qi Lei, Jianmeng Lv, Bei Kang, Hena Guo, Yulang Fei, Ruili Chen, Hui Guo, Qian Yang

**Affiliations:** ^1^Department of Neurology, Shaanxi Provincial People's Hospital, Xi'an, China; ^2^Department of Clinical Medicine, Xi'an Medical University, Xi'an, China

**Keywords:** lateral medullary syndrome (LMS), Opalski syndrome, SUNCT syndrome, medullary infarction, headache, stroke, trigeminal autonomic cephalalgias (TAC), Horner's syndrome

## Abstract

Opalski syndrome is a rare variation of lateral medullary syndrome (LMS) accompanied by ipsilateral hemiparesis. Short-lasting unilateral neuralgiform headaches with conjunctival injection and tearing (SUNCT) is a rare headache syndrome which belongs to the trigeminal autonomic cephalalgias. SUNCT syndrome has been previously described in association with LMS. We here describe a case of SUNCT syndrome with Opalski syndrome caused by dorsolateral medullary infarction.

## Background

Lateral medullary syndrome (LMS) (Wallenberg syndrome), is also called posterior inferior cerebellar artery syndrome. It is an uncommon stroke. Opalski syndrome is a rare variant of LMS, with ipsilateral hemiplegia (1, 2). Short-lasting unilateral neuralgiform headaches with conjunctival injection and tearing (SUNCT) is a rare headache disorder. It belongs to the trigeminal autonomic cephalalgias (TAC). Most of secondary SUNCT cases are associated with posterior fossa abnormalities (3). Hereby, we report a case of SUNCT syndrome with Opalski syndrome caused by dorsolateral medullary infarction. We described the clinical manifestations, imaging findings, treatment, and outcomes.

## Case Presentation

A 44-year-old Chinese male was admitted to our hospital with headache, dizziness, nausea, and vomit for 6 days, and right limbs weakness for 2 h. He felt tearing pain on the right face and right temporal occipital. The pain most of time lasted about 10–180 s, less than 300 s. The headache frequency was more than 10 times per day. He had right drooping eyelid and right face decreased sweating, accompanied by right-sided redness of eye, lacrimation, and nasal congestion. He also felt numbness of right face and left half of his body and limbs, paraesthesia in right side of body, difficulty in swallowing, dysarthria, hiccups, hoarseness, and unsteady gait with multiple episodes of falls always to his right side. He had no history of diabetes, hypertension, or coronary artery disease. There was no history of trauma in the past. He never smoked or consumed alcohol. His family history was not significant.

**Figure 1:**
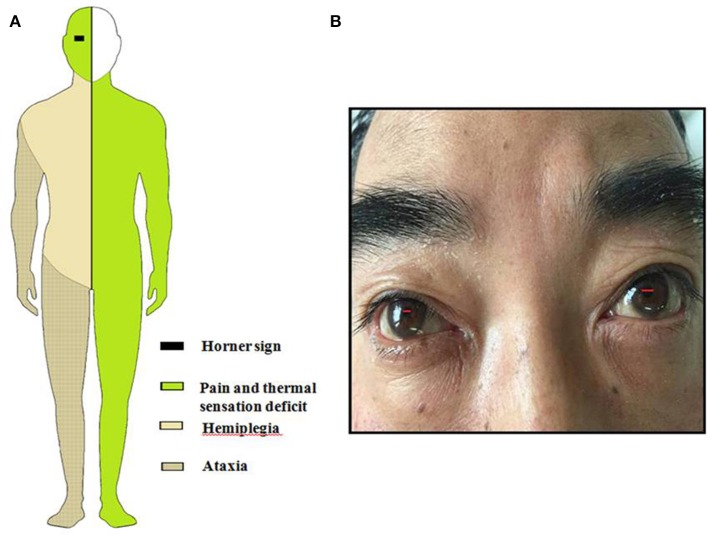
**(A)** Schematic drawing of Horner sign, pain, and thermal sensory deficit, ataxia and hemiparesis. **(B)** This figure shows patient's lacrimation and conjunctival injection in the right eye associated with Horner's syndrome.

**Figure 2:**
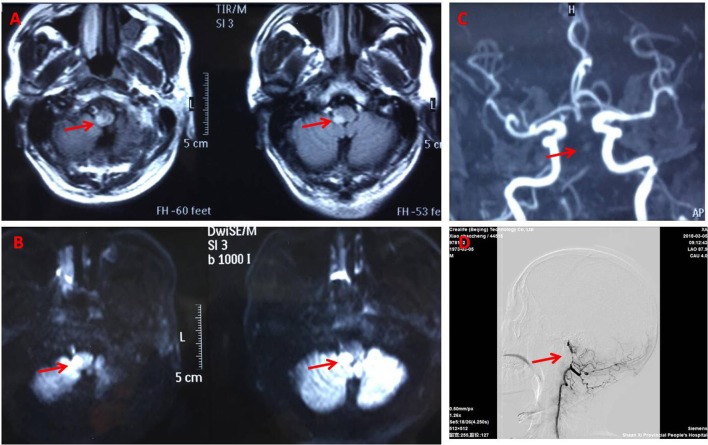
Brain magnetic resonance imaging. Fluid attenuated inversion recovery **(A)** and Diffusion weighted **(B)** magnetic resonance showing the right dorsolateral medullary ischemic infarction extending to the upper cervical cord. Magnetic resonance angiography **(C)** and DSA **(D)** (arrowhead) suggest basillar artery stenosis.

General examination, vital parameters (T, P, BP, R), cardiovascular, respiratory, and abdominal examinations were unremarkable. His blood pressure was found to be 100–120/56–85 mmHg, and his heart was in normal sinus rhythm. Neurological examination revealed that he was somnolent. Right Horner's syndrome (ptosis, myosis, and anhydrosis) and clockwise rotational nystagmus were detected. His gag reflex was reduced. He had right-sided hemiparesis, muscle strength was diminished, MRC (Medical Research Council) grade muscle strength was 4/5 power and Babinski sign was present on the right. Right limbs showed incoordination and there was loss of pain and temperature sensation on the right side of his face and left side of his body. The finger-nose test and heel-knee-tibia test revealed ataxia on the right side of his body. Neurological findings suggested that right hemiparesis accompanied the typical findings of LMS (Figure 1). The rest of the exam was unremarkable. Laboratory blood tests showed no other abnormalities except mildly elevated levels of urea and creatinine. No pathological findings were detected in computed tomography of the brain. DWI of MR imaging of the brain showed acute ischemic infarction of the right dorsolateral medulla. The area of infarction extends anteriorly within the medulla. Oxygen (2–3 L/min) was delivered through a T-piece in tracheostomy. rt-PA was administered in compliance with the standard dose, 0.9 mg/kg. Then antiplatelet treatment (asprin and clopidogrel) and statin were initiated. The cranial and cervical magnetic resonance angiography (MRA) revealed advanced stenosis in the basilar artery and right vertebral artery (Figure 2). Further digital subtraction angiography (DSA) confirmed this (Figure 2). Vertebral artery dissection (VAD) and aneurysms were excluded.

The patient gradually improved and was discharged with remarkable improvements after 2 weeks after conventional treatment for lateral medullar infarction. He had a 90% improvement in pain intensity and frequency from brief pain inventory. He was fully conscious without diplopia and nystagmus, eating by himself, and walking independently for a short distance, although unsteady. The symptoms of headache resolved 1 month later. After 5 months, an MRI brain scan and a MRA of the cerebral circulation were performed for follow up. High resolution MR images of the wall of the vertebrobasilar artery showed localized stenoses of both vertebral arteries due to unstable appearing atheromatous plaque (Figure 2). One year later the patient still had some residual neurological symptomatology, including a mild deficit in pain and temperature sensation on the right side of his face and left side of body, mild dizziness, right face anhydrosis, mild right limbs numbness, and widened step base. He could walk independently and even run a little bit.

## Discussion

Our patient presented with not only classical symptoms of LMS but also with right-sided hemiparesis, and right limb incoordination. The classical presentation of LMS consists of crossed sensory deficits, specifically loss of pain and temperature sensation affecting ipsilateral facial, trunk, and contralateral extremities (4). Other features include vertigo, nystagmus, hoarseness, dysphagia, ipsilateral cerebellar signs, and Horner's syndrome. The occurrence of LMS in a patient presenting with hemiparesis suggests either an Opalski syndrome or a Babinski-Nageotte syndrome (5–7). Our patient presents Opalski syndrome.

The mechanisms of ipsilateral hemiparesis can be explained in the following ways. Firstly, ipsilateral hemiparesis is attributed to the involvement of corticospinal fibers caudal to pyramidal decussation due to the extension of the ischemia from the lateral medulla to the upper cervical cord and anterior extension within medulla (8, 9). Secondly, medullary penetrating arteries rising from anterior spinal artery or distal vertebral artery supplying the pyramidal fibers post decussation may be involved (10). Thirdly, the involvement of pyramidal fibers may be in the border-zone area between the anterior and posterior spinal arteries, and fail to get enough blood supply due to artery occlusion or stenosis (11). Lastly, some people think hemiparesis may be a result of spinocerebellar hypotonic syndrome (12). A study using diffusion tensor imaging techniques and superimposed images to generate a directionally encoded color map, suggested Opalski syndrome may arise as a consequence of ipsilateral corticospinal tract involvement after the pyramidal decussation, or compression of the decussation (9).

Another unusual sign of our patient was headache at the onset of symptoms. He had paroxysmal severe short lasting unilateral right-sided temporal pain accompanied by right-sided redness of eye, lacrimation, and nasal congestion. He also had right ptosis, miosis, and right face anhydrosis. The patient was concomitantly diagnosed with SUNCT according to the criteria in the International Classification of Headache Disorders, 3rd edition (beta version) (ICHD-3beta) (13). SUNCT is a rare headache disorder. Sjaastad first proposed a case of symptomatic SUNCT syndrome in 1989 (14). It is characterized by unilateral paroxysmal headache accompanied by autonomic manifestations such as conjunctival injection, lacrimation, nasal stuffiness, and rhinorrhoea.

Brain MRI and DWI of this patient revealed right dorsolateral medulla acute ischemic infarction. The symptoms of SUNCT resolved after conventional treatment for lateral medullar infarction but right face anhydrosis and sensory deficiency were still present. It seems that there is an association between the symptoms of acute dorsolateral medulla infarction and SUNCT. There are some features enabling differentiation between paroxysmal severe headache of our case and classical SUNCT. First, paroxysmal severe headache with Horner's syndrome (ptosis, myosis, and anhydrosis) contrasts to classical SUNCT. The patient might have had a persistent sympathetic injury. Moreover, the patient had both right face sensory deficits and paroxysmal severe headache, as one of the distinguishing features from classical SUNCT.

Several previous reports showed that most of secondary SUNCT are associated with posterior fossa abnormalities, such as epidermoid tumor or vascular malformation (15), neuromyelitis optica and relapsing progressive multiple sclerosis (MS) (16). SUNCT syndrome and presumed brainstem infarction has been reported by Penart et al. and Jin et al. (17, 18). Headaches in both cases were resolved after several weeks with no specific drugs such as gabapentin, Topamax, or lamotrigine. Lambru et al. (19) reported a case of coexisting chronic SUNCT and TN-like phenotypes caused by haemorrhagic infarct of the dorsolateral medulla due to the PICA dissection, suggesting that vascular insults of the brainstem can exacerbate a SUNCT-like syndrome. A few papers have reported that headache resembling trigeminal autonomic cephalalgias (TACs) can be induced by dorsolateral medullary ischemic infarction (20). Some studies confirmed hypothalamic involvement in TACs' pathophysiology (21). The hypothalamospinal tract lies in dorsolateral medulla. In the classic pain transmission pathway, pain afferents from the trigeminovascular system synapse in the trigeminocerivcal complex, with projections to the thalamus, resulting in activation of cortical areas (22). In our patient, the acute medullary infarction was associated with subsequent SUNCT. The mechanism of this secondary SUNCT might differ from classic primary SUNCT. Ischemic lesions of the medulla may stimulate the trigeminal nucleus.

## Conclusion

Opalski syndrome is a rare variant of LMS with concomitant ipsilateral hemiparesis. Secondary SUNCT syndrome is rarely caused by dorsolateral medullary infarction. We reported a case of Opalski syndrome and SUNCT syndrome occurring simultaneously. The mechanism for this is perhaps due to ischemia extending anteriorly within the medullar and inferiorly to involve caudal corticospinal fibers after pyramidal decussation, and that stimulation of the trigeminal nucleus leads to aberrant activation of trigeminovascular system.

## Ethics Statement

The studies involving human participants were reviewed and approved by Shaanxi Provincial People's Hospital, Xi'an, Shaanxi, China. The patients/participants provided their written informed consent to participate in this study. Written informed consent was obtained from the individual(s) for the publication of any potentially identifiable images or data included in this article.

## Author Contributions

QL studied concept, carried out the treatment, and drafting the manuscript. JL involved in the conception, design, and analysis. BK and HeG involved in the interpretation of data. YF involved in drafting the manuscript. RC and HuG involved in drafting figure. QY revised the manuscript. All authors read and approved the final manuscript.

### Conflict of Interest

The authors declare that the research was conducted in the absence of any commercial or financial relationships that could be construed as a potential conflict of interest.
